# Molecular and phenotypic characterization of methicillin-resistant *Staphylococcus aureus* isolates from a tertiary hospital in the Philippines

**DOI:** 10.1186/s41182-016-0003-z

**Published:** 2016-03-14

**Authors:** Demetrio L. Valle, Phyllis Anne P. Paclibare, Esperanza C. Cabrera, Windell L. Rivera

**Affiliations:** Institute of Biology, College of Science, University of the Philippines, Diliman, Quezon City, 1101 Philippines; Department of Pathology and Laboratories, Makati Medical Center, Makati City, 1229 Philippines; Natural Sciences Research Institute, University of the Philippines, Diliman, Quezon City, 1101 Philippines; Biology Department, De La Salle University, Taft Ave., Manila City, 1004 Philippines

**Keywords:** *Staphylococcus aureus*, Methicillin-resistant *S. aureus* (MRSA), SCC*mec*, Panton-Valentine leukocidin (PVL), Phylogram, Random amplified polymorphic DNA (RAPD), Philippines

## Abstract

**Background:**

Methicillin-resistant *Staphylococcus aureus* (MRSA) poses a major threat to public health worldwide. There are relatively few studies addressing the molecular epidemiology of MRSA in the Philippines.

**Methods:**

This study characterized MRSA isolates in terms of their antimicrobial susceptibility profile, the SCC*mec* type, and the presence of *lukF*-*lukS* genes for Panton-Valentine leukocidin (PVL) and determined the relatedness of the isolates by random amplified polymorphic DNA (RAPD)-polymerase chain reaction (PCR).

**Results:**

A total of 236 *S. aureus* were isolated from clinical specimens of the Makati Medical Center in Makati City, Philippines, between January 2013 and June 2013, and 108 or 45.76 % were found to be MRSA. Results showed that the MRSA strains were resistant to trimethoprim-sulfamethoxazole (20.37 %), azithromycin (10.19 %), gentamicin (5.56 %), and linezolid (4.63 %), while all were susceptible to vancomycin, nitrofurantoin, levofloxacin, minocycline, rifampin, and tetracycline. One isolate was found positive for inducible clindamycin resistance. All of the 108 MRSA strains were confirmed to carry the *mecA* and SCC*mec* genes, while the PVL genes were detected in 41 (38 %) of the isolates. Ninety-six isolates (89 %) carried SCC*mec* type IV, while the remaining isolates carried SCC*mec* type I (11 isolates) or type III (one isolate).

**Conclusion:**

This study is the first to present a comprehensive MRSA surveillance data with molecular characterization in a tertiary hospital in the Philippines.

## Background

*Staphylococcus aureus* is the most potent and significant species of staphylococci that causes infections from simple furuncles (boils) and carbuncles to deadly necrotizing pneumonia and toxic shock syndrome [1]. It causes considerable morbidity and mortality in both healthcare and community settings despite enormous advances in medical care. In the 1960s, an *S. aureus* strain resistant to the then newly introduced antibiotic methicillin, thus called methicillin-resistant *S. aureus* (MRSA), emerged as a significant pathogen in hospitals and intensive care units, especially in crowded facilities [2, 3]. Since then, MRSA strains have developed resistance to a variety of other antimicrobial agents and are the principal causes of hospital-acquired infections worldwide [4].

Methicillin is a β-lactam antibiotic that is resistant to the action of β-lactamase secreted by many penicillin-resistant bacteria [5]. It acts via competitive inhibition of transpeptidase enzyme by its affinity to penicillin-binding protein 2 (PBP2) used by bacteria to cross-link the peptide (d-alanyl-alanine) mandatory for peptidoglycan synthesis. It was developed to treat staphylococcal infections. Resistance to methicillin is developed due to acquiring a penicillin-binding protein 2A (PBP2A) encoded by the *mecA* gene from a mobile “staphylococcal cassette chromosome (SCC) *mec*.” As of 2009, 11 types of SCC*mec*, which differ in the combination of the type of *ccr* gene complex and the class of *mec* gene complex present in the cassette, have been identified in *S. aureus* and in *Staphylococcus epidermidis* [4, 6]. The current diagnosis for MRSA includes resistance to either oxacillin or cefoxitin, which indicate non-susceptibility to all categories of β-lactams and cephamycins, except anti-MRSA cephalosporins [7].

MRSA infections have been acknowledged for several years as either healthcare-associated (HA-MRSA) or community-acquired (CA-MRSA) [8, 9]. Unlike the HA-MRSA, it is alarming that CA-MRSA infections are common in healthy individuals with no or partial exposure to healthcare facilities and in a variety of populations, which include the prisoners, children, adolescents, and athletes [10]. Outbreak and in-depth analysis of CA-MRSA were first reported by Udo et al. [11] among indigenous Australians in Western Australia without any healthcare contacts. CA-MRSA generally causes soft tissue and primary skin infections. Furthermore, CA-MRSA abscess outbreaks were reported among people in closed living communities like jail inmates, military recruits, football players, and homosexual men [12, 13].

CA-MRSA strains were shown to have distinctive genetic makeup, antimicrobial profiles, and virulence properties that set them apart from HA-MRSA strains [14]. The strong relationship between CA-MRSA and Panton-Valentine leukocidin (PVL) is demonstrated by the presence of the genes *lukF*-*lukS* only in CA strains [15]. PVL is an exotoxin that acts by destroying the leukocytes via formation of pores which causes higher cation permeability of the cell membrane. It is usually present in USA 300 and USA 400 strains and harbored by SCC*mec* IV-containing strains [16]. PVL is known to induce necrosis and is associated with necrotizing pneumonia, furunculosis, cutaneous abscess, soft tissues, and invasive skin infections [13, 17, 18]. However, for the past decade, HA/CA distinctions have not been consistent as CA strains become “domesticated” to the healthcare settings [19] and HA strains become “feral” and establish in the community [20]. Due to the molecular variation of MRSA, some authors have suggested that antimicrobial susceptibility may continue to be a distinguishing trait of CA-MRSA [21].

Many phenotypic and molecular techniques are available to differentiate MRSA isolates. The most common phenotypic method is antimicrobial susceptibility testing (AST); however, isolates with different genetic profiles may have the same antibiogram patterns [22]. This limitation of phenotypic methods has elicited the development of molecular or DNA-based procedures, including restriction fragment length polymorphism (RFLP) analysis, ribotyping, binary typing, and PCR-based methods [23–26]. For long-term and broad epidemiological studies of MRSA, a number of research deal with either multilocus sequence typing (MLST) or pulse-field gel electrophoresis (PFGE) [22, 27]. These methods allow for simultaneous amplification of multiple regions of the DNA resulting in distinct patterns, in contrast to other targeted techniques such as staphylococcal protein A (spa) or direct repeat unit (dru) typing [28]. However, MLST is costly, lengthy, and labor intensive for clinical use while PFGE is subjected to biased interpretation in comparing patterns and fingerprint images [22].

Another useful method is random amplified polymorphic DNA (RAPD) typing, which is based on the genomic DNA amplification using a single short oligonucleotide primer of arbitrary or random sequence [29]. PCR products are varied due to random hybridization of primers with DNA sequences that vary among strains [30]. Due to its versatility, easy procedure, and high level of discrimination, RAPD is widely used in epidemiological studies of HA-MRSA [31]. It is critical to have a precise epidemiological typing of MRSA so that it would be easier to control the spread of infection and track epidemics despite the inherent variation of strains. The challenge today is to keep on making bacterial databases linking genetic markers and clinical outcomes so that important correlations of the disease can be detected. This study then aimed to investigate the molecular diversity of MRSA isolates obtained from out- and inpatients of Makati Medical Center in Makati City, Philippines, between January 2013 and June 2013 using RAPD.

## Methods

### Bacterial isolates

This study included all clinical isolates submitted for culture and sensitivity at the Department of Pathology and Laboratories, Makati Medical Center, from January 1, 2013 to June 30, 2013. *S. aureus* isolates were identified using Vitek^®^ 2 (bioMérieux, Marcy-l’Etoile, France) GP colorimetric identification card. Resistance to oxacillin or cefoxitin was confirmed by Vitek^®^ 2 AST-GP67-22226 (bioMérieux, Marcy-l’Etoile, France), with resistance defined as oxacillin minimum inhibitory concentration (MIC) ≥4 μg/ml and cefoxitin MIC ≥8 μg/ml. Staph MRSA^TM^ latex agglutination test (bioMérieux, Marcy-l’Etoile, France) was performed for PBP2A determination. In the case of multiple *S. aureus* isolates from the same patient, the first detected resistant specimen was included in the study. The isolates were stored at −80^o^C and were subcultured on nutrient agar slants prior to DNA extraction.

### Antimicrobial susceptibility testing

In addition to oxacillin and cefoxitin, other antimicrobials listed for routine reporting for *S. aureus* were included for AST using Vitek^®^ 2 AST-GP67-22226 (bioMérieux, Marcy-l’Etoile, France) test card for Gram-positive susceptibility. The MIC interpretive standard or breakpoint values were set following the guidelines of Clinical and Laboratory Standard Institute M100-S23 [32]. *S. aureus* ATCC^®^ 25923 was included as control. Isolates resistant to erythromycin and susceptible to clindamycin were screened for inducible clindamycin resistance using modified disk diffusion test or D-zone test.

### DNA extraction

Genomic DNA from MRSA isolates was extracted following the rapid boiling method described by Zhang et al. [33]. One to five isolated colonies from a nutrient agar (NA) plate were suspended in 50 μl sterile water and heated at 99 °C for 10 min. The suspension was pelleted by centrifugation at 20,000×*g* at 4 °C. The supernatant was collected and stored at −20 °C until further use. DNA yield was confirmed by NanoDrop™ spectrophotometry prior to PCR-based assays.

### PCR amplification of *mecA* and PVL genes

Multiplex PCR assay for the simultaneous identification of *mecA*, *lukF*-*lukS*, and 16S ribosomal RNA (rRNA) genes was performed following the method described by Zhang et al. [33] and McClure et al. [34]. Amplification of 16S rRNA served as an internal control to rule out any amplification inhibitors. Other targets include *mecA*, which is a determinant of MRSA, and *lukF*-*lukS*, which encodes the F and S biocomponent proteins of PVL. The primers are listed in Table 1. The thermocycling conditions were based on McClure et al. [34] with a final extension modification at 72 °C for 10 min. PCR-based products in the entire study were separated in 1.8 % agarose gel and were analyzed using UV transilluminator after staining with ethidium bromide.

**Table 1:** Primers for the detection of *mecA*, *lukF*-*lukS*, and 16S rDNA for *Staphylococcus aureus*

Primer	Nucleotide sequence (5′ to 3′)	Product size (bp)
Staph 756F	AACTCTGTTATTAGGGAAGAACA	756
Staph 750R	CCAACCTTCCTCCGGTTTGTCACC	
Luk-PV-1	ATCATTAGGTAAAATGTCTGGACATGATCCA	433
Luk-PV-2	GCATCAAGTGTATTGGATAGCAAAAGC	
MecA1	GTAGAAATGACTGAACGTCCGATAA	310
MecA2	CCAATTCCACATTGTTTCGGTCTAA	

### PCR amplification of SCC*mec* type

All isolates were subjected for multiplex PCR for the detection of six SCC*mec* types (I, IA, II, III, IV, IVA) based on loci located upstream and downstream of the *mecA* gene as described by Zetola et al. [35]. The primer sequences are given in Table 2. The *mecA* gene was included to serve as internal control. The multiplex PCR was performed following the method of Cabrera et al. [12].

**Table 2:** Primers for the determination of SCC*mec* type of MRSA isolates

Primer	Nucleotide sequence (5′ to 3′)	Product size (bp)	SCC*mec* type/region
CIF2 F2	TTCGAGTTGCTGATGAAGAAGG	495	I, IA
CIF2 R2	ATTTACCACAATTACTACCAGC		
KDP F1	AATCATCTGCCATTGGTGATGC	284	II
KDP R1	CGAATGAAGTGAAAGAAAGTGG		
DCS F2	CATCCTATGATAGCTTGGTC	342	I, IA, II, IV, IVA
DCS R2	CTAAATCATAGCCATGACCG		
RIF4 F3	GTGATTGTTCGAGATATGTGG	243	III
RIF4 R9	CGCTTTATCTGTATCTATCGC		
IS431 P4	CAGGTCTCTTCAGATCTACG	381	IA, II, IVA
PUB110 R1	GAGCCATAAACACCAATAGCC		
MECA P4	TCCAGATTACAACTTCACCAGG	162	*mecA* gene
MECA P7	CCACTTCATATCTTGTAACG		

### Random amplified polymorphic DNA typing and analysis

RAPD typing was performed following the method described by Casey et al. [36] utilizing the 10-mer primer 5′-AGC GTC ACT G-3′, with few modifications. The primer has been used in characterizing strains of *S. aureus* [36, 37]. DNA sizing and quantifications were done using Agilent 2100 bioanalyzer (Agilent technologists, USA). RAPD fingerprints were analyzed using the Sequentix—Digital DNA Processing (Germany). The lanes of the gel images were extracted using GelQuest (Sequentix, Germany) and were transformed into a trace data curve. Peaks were compared with base sizes from 110 to 1000 bp, and hyperbin width was 10 bp. The following parameters were taken: base sizes, peak heights, areas, and area-to-height ratio, and a hyperbin table (01-matrix) was obtained, which can be used for binary analysis using ClusterVis (Sequentix, Germany). The Jaccard distance measure was used for cluster analysis. The Jaccard coefficient (or similarity) is calculated from datasets where the presence and absence of characters between two or more samples are compared. Such a dataset consists of 1s for the presence of a certain character in a sample and 0s for the absence of a character which is also called a binary matrix. The resulting distance matrix was analyzed using the neighbor-joining (NJ) algorithm. Three *S. aureus* strains (SA, Sab, and SAc) were used as controls, while two *Escherichia coli* samples (EC and ECb) were used as outgroup for rooting the tree. The bacterial genetic relationships are shown graphically as a phylogram to show the topology and the real distances between the samples.

## Results

### Study population and prevalence of MRSA

Of the 236 *S. aureus* isolates, 108 were found to be MRSA (45.76 %). Patients were from ages 5 months to 100 years old, and the mean age was 36.9 ± 22.4 (mean ± SD) years. The MRSA-positive subjects included 66 (61.1 %) males and 42 (38.9 %) females. Isolates were collected from 66 outpatients (61.1 %) and 42 inpatients (38.9 %). Among the 42 inpatients, 21 (50 %) were identified with clinical infections within 48 h of hospital admission. The MRSA isolates were from the skin, wound, superficial abscess (79 cases), respiratory tract specimens (14 cases), blood (6 cases), ear/eye discharge (4 cases), and deep abscess (5 cases).

### Antimicrobial susceptibility pattern

The antimicrobial susceptibility patterns of all MRSA isolates are summarized in Table 3. As expected, all MRSA isolates were resistant to the ß-lactams penicillin, oxacillin, and cefoxitin. Resistance was not observed in tetracycline, minocycline, rifampin, and vancomycin. For the rest of the antimicrobials, resistance was low. The susceptibility of the isolates to vancomycin and linezolid was also expected as these drugs are currently effective against MRSA.

**Table 3:** Antimicrobial susceptibility profile of MRSA isolates

Antimicrobial	Susceptible	Intermediate	Resistant
Penicillin	0	0	108 (100 %)
Oxacillin	0	0	108 (100 %)
Cefoxitin	0	0	108 (100 %)
Ciprofloxacin	100 (92.59 %)	4 (3.70 %)	4 (3.7 %)
Levofloxacin	99 (91.67 %)	9 (8.33 %)	0
Clindamycin	99 (91.67 %)	6 (5.56 %)	3 (2.78 %)
Erythromycin	102 (94.44 %)	3 (2.78 %)	3 (2.78 %)
Gentamicin	102 (94.44 %)	0	6 (5.56 %)
Minocycline	108 (100 %)	0	0
Tetracycline	108 (100 %)	0	0
Vancomycin	108 (100 %)	0	0
Azithromycin	86 (79.63)	11 (10.19 %)	11 (10.19 %)
Nitrofurantoin	54 (50.00 %)	54 (50.00 %)	0
TMP-SXT	82 (75.93 %)	4 (3.70 %)	22 (20.37 %)
Rifampin	108 (100 %)	0	0
Linezolid	103 (95.37 %)	0	5 (4.63 %)

### Detection of 16S rRNA, *mecA*, and PVL genes

Both the internal control 16S rRNA for *Staphylococcus* spp. and *mecA* genes were present in all MRSA isolates. Of the 108 MRSA isolates investigated, 41 (38.0 %) were positive for PVL genes. Table 4 shows the distribution of the PVL genes among different types of clinical samples. Twenty-four (58.5 %) cases were from outpatients and 17 (41.5 %) cases were from inpatients. The other samples positive for PVL genes include wound; wound discharge; abscess; respiratory tract samples; blood; bile; and eye, mouth, and ear discharge. It was noted that isolates from two patients who died from septicemia were found to be positive for PVL.

**Table 4:** Carriage of the PVL genes *lukF*-*lukS* among MRSA isolates

Clinical sample	Outpatient	Inpatient	Total
Wound, wound discharge, abscess	22 (91.6 %)	11 (64.7 %)	33 (80.4 %)
Respiratory tract	1 (4.2 %)	1 (5.9 %)	2 (4.9 %)
Blood, bile	1 (4.2 %)	3 (17.6 %)	4 (9.8 %)
Eye, mouth, ear discharge	0	2 (11.8 %)	2 (4.9 %)
Total	24 (58.5 %)	17 (41.5 %)	41

### Determination of SCC*mec* type

**Fig. 1:**
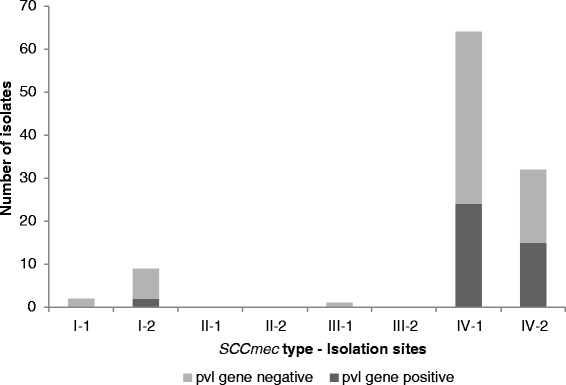
Prevalence of SCC*mec* types at the Makati Medical Center from January to June 2013. Isolation type *1* denotes samples isolated from outpatients while *2* is samples isolated from inpatients. *I*, *II*, *III*, and *IV* mean SCC*mec* types I, II, III, and IV, respectively

Ninety-six out of the 108 MRSA isolates (89 %) carried SCC*mec* type IV. Eleven isolates carried SCC*mec* type I, only one carried SCC*mec* type III, and none for type II. The majority of the MRSA strains came from a variety of samples. The SCC*mec* type I strains were isolated from wound, blood samples, and tracheal aspirates, while the type III strain came from a thigh abscess. Figure 1 shows the distribution of the detected SCC*mec* types among different sample types. Aside from the majority (39/41) of PVL carriers among the type IV, 2 PVL carriers belong to type I, both from blood samples.

### RAPD analysis

**Fig. 2:**
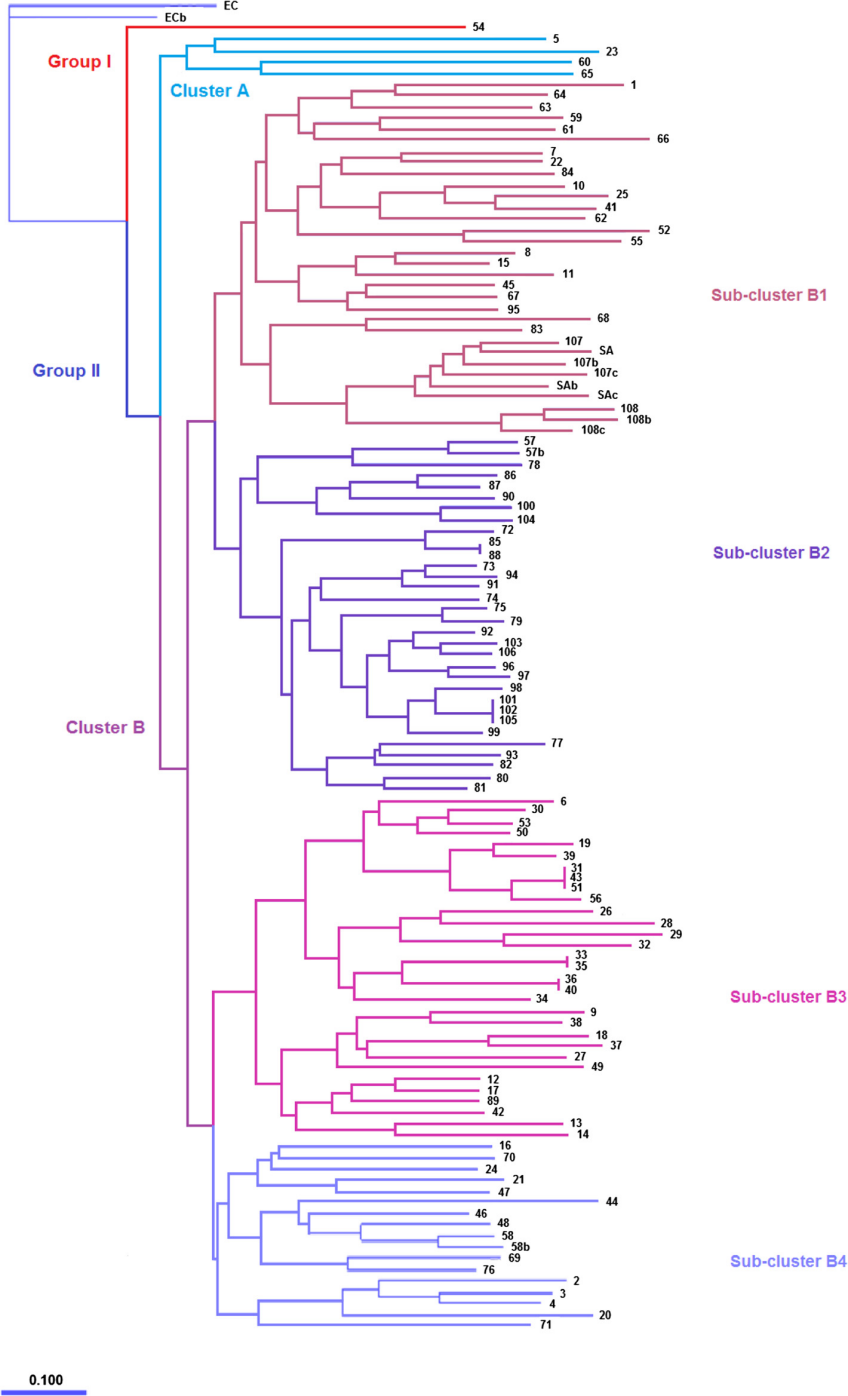
Phylogram of genetic relationships between 108 isolates of *S. aureus* by RAPD

RAPD typing yielded 1–6 distinct bands per isolate, revealing DNA markers ranging from 100 to 7000 bp. The phylogram (Fig. 2) obtained resulted in two main groups, group I and group II, which further branched into two main clusters (A and B). The two *E. coli* samples (EC and ECb) are reference strains and were used as outgroup for rooting the tree. Group I has only one isolate (Isolate 54). Isolate 54 is distinct from all the other isolates since it is the only isolate with just a single prominent band. Isolate 54 was taken from a wound discharge from an outpatient. It is one of the two isolates with SCC*mec* type I from outpatients and does not carry the PVL genes. Group II cluster A consisted of four isolates, all from patients confined in the hospital, expressing both the SCC*mec* type IV and the PVL genes. Group II cluster B has four sub-clusters, B_1_, B_2_, B_3_, and B_4_. Sub-cluster B_1_ contained 25 isolates; all of them came from outpatients with SCC*mec* type IV. The PVL genes were present only in four isolates, with one from an outpatient sample. The *S. aureus* strains (SA, Sab, and SAc) also clustered in this group as well as the triplicate samples of isolates 107 and 108. Sub-cluster B_2_ is comprised of 31 isolates from both outpatients and inpatients, with 6 out of the 31 isolates that carry SCC*mec* type I, and one carries SCC*mec* type III. Of the 10 PVL gene carriers with SCC*mec* type IV, seven came from outpatients and three from inpatients; while two out of six SCC*mec* type I isolates carried the PVL genes, both from outpatients. The sub-cluster B_3_ also consisted of 31 isolates, including 14 PVL gene carriers with SCC*mec* type IV, nine from outpatients and five from inpatients, and two inpatient isolates with SCC*mec* type I, without the PVL genes. Finally, sub-cluster B_4_ contained 16 isolates, including 15 that carried SCC*mec* type IV and one with SCC*mec* type I. The PVL genes were detected in seven isolates with SCC*mec* type IV in this group, five from outpatients and two from inpatients. The phylogram obtained showed that the SCC*mec* type and PVL genes are widely distributed. Only cluster A with the smallest population showed similarities in all parameters, which include the source of sample, presence of PVL genes, and SCC*mec* type IV. The method produced 45 major banding patterns in total.

## Discussion

This study is the first to present a comprehensive MRSA surveillance data with molecular characterization in a Philippine tertiary hospital. This current study revealed a prevalence of 108 out of 236 (45.76 %) MRSA among clinical isolates received. This prevalence is comparable to previously reported studies from different countries, 22–57 % in the USA, 43–58 % in Italy, 54 % in Portugal, 42–44 % in Argentina and Paraguay, 48 % in Mexico, and 44 % in Greece. Lower rates were reported in other European countries, 0.5 % in Iceland, 2 % in Switzerland and Netherlands, and 7.8 % in Italy [38–43]. In the Philippines, few studies showed lower prevalence rates, 18 % in three tertiary hospitals in Manila from 1999 to 2003 and 13.6 % in a medical center in Quezon City in 2001 [44, 45].

Susceptibility testing of MRSA may be challenging because of the heterogeneous resistance displayed by many isolates. In this study, resistance to trimethoprim-sulfamethoxazole (SXT) was observed in 22 cases (20.37 %) from the outpatients, while no resistance was observed for tetracycline, minocycline, rifampin, and vancomycin. Contrastingly, antibiogram patterns from previous studies showed high resistance to tetracycline, clindamycin, and erythromycin and low resistance to SXT among CA-MRSA strains in Taiwan and HA-MRSA strains from Czech Republic [46, 47]. High resistance to levofloxacin (98.6 %), erythromycin (97.8 %), tetracycline (94.2 %), and rifampin (87.1 %) were also reported in China [48]. These conflicting patterns may be brought about by geographical differences and also by the nature of host or sample type, thus making a universal differentiation of MRSA strains a challenge. Antibiograms, however, may be important in monitoring the current and emerging resistance patterns in MRSA. While there have been reports of MRSA resistant to vancomycin and linezolid [49, 50], the fact that these drugs remained effective against MRSA strains evaluated in this study was reassuring.

The PVL genes, *lukF*-*lukS*, were detected in 41 cases (38.0 %) of MRSA isolates. The prevalence of the PVL genes in this study was unexpectedly higher than reported studies in other foreign hospitals. Some studies from Czech Republic, China, Trinidad and Tobago, and Austria and Hungary reported 0 % prevalence of PVL genes in all confirmed MRSA isolates [47, 48, 51, 52]. PVL is a cytolytic toxin comprised of F and S subunits which are encoded by the *lukPV* operon, which contains the *lukF*-*lukS* genes. It forms pores exclusively in leukocytes and induces high inflammatory mediators’ response such as leukotriene B4, IL-8, and histamine [53]. Presently, PVL gene-positive MRSA strains are responsible for nosocomial infections in countries where the prevalence of CA-MRSA is high such as the Philippines [12]. PVL has been thought to be a good marker for CA-MRSA since it has been reported to be high among these isolates [54]. However, it is postulated that CA-MRSA strains are now beginning to infiltrate hospital settings making it impossible to be differentiated from HA-MRSA strains. Similarly, this present study was able to detect PVL gene carriers among out- and inpatients.

Typing of MRSA isolates within the context of epidemiological investigation of nosocomial and community-acquired infections is of great significance for considering the connection and similarities of isolates, for establishing the series of infections, and, accordingly, for the application of suitable infection control and preventive measures. Clonal relatedness can only be deduced from molecular studies comparing isolates from MRSA strains. The RAPD typing done has allowed the genotyping of the *S. aureus* isolates. The fingerprints created by this primer revealed distinctive profiles for every strain in terms of number and banding patterns. There was a definite clustering of the isolates into at least seven large clades, which indicate that the primers have resolved the strain types into at least seven groups. Group I, group B cluster A, and sub-cluster B_1_ show good distinguishing traits than the other clades. Group I separated one (Isolate 54) SCC*mec* type I from an external wound discharge from the rest. Then, group II cluster A included inpatient isolates only with SCC*mec* type IV and the PVL genes. This clade may reflect the so-called domesticated strains that circulate in community settings but might be isolated from hospital patients [55]. Sub-cluster B_1_ also show clustering of non-PVL gene carriage among its members. However, it is to note that most of the isolates included here came from outpatients. The close topology of the latter clades may support the domestication of CA-MRSA strains, as these commonly lose functional PVL when infected patients remain in hospital settings for a time period [19].

The RAPD analysis in this study, however, was not able to establish the correlation of the characteristics of the isolates based on the clinical specimen source, SCC*mec* types, and the incidence of the PVL genes. Sub-clusters B_2_, B_3_, and B_4_ contained random clustering of isolates from in- and outpatients; presence and absence of PVL genes; and a combination of SCC*mec* types I, III, and IV. This may be due to chance accrual of neutral mutations or to alteration of the predominant strain to adapt to constant environmental changes. Therefore, these could not be traced as either HA- or CA-MRSA. Mutations of a single strain for a period of time provide further avenues for the procurement of pathogenic features such as antimicrobial resistance, and thus, genetic distinction offers rise to broad genomic and phenotypic array [56]. Furthermore, the presence of the PVL genes did not associate with the outpatients or CA-MRSA, since only 17 of the 41 outpatients carried the gene. In addition to the possible domestication of the CA strains, the study covered a 6-month period, long enough for increasing the number of polymorphisms for such MRSA strains [55].

## Conclusions

MRSA remains one of the most important threats to public health due to the fast dissemination and diversification of MRSA strains with greater virulence and antimicrobial resistance. In the Philippines, MRSA is a principal source of nosocomial infections, and the prevalence of MRSA in community-acquired infections is mounting. Categorization of these clones is significant if suitable local treatment plans are to be established. A comprehensive investigation of strains disseminating within an area may be used to evaluate the association between clonal types, disease symptoms, antimicrobial choice, and clinical outcomes. Hence, the evidence gathered from typing *S. aureus* isolates can assist the infection control teams to recognize the epidemiology of this organism in the hospitals and communities and furthermore help in the application of effective control measures, at least in Philippines.
